# Late Post-Conditioning with Sevoflurane after Cardiac Surgery - Are Surrogate Markers Associated with Clinical Outcome?

**DOI:** 10.1371/journal.pone.0132165

**Published:** 2015-07-21

**Authors:** John M. Bonvini, Beatrice Beck-Schimmer, Sonja J. Kuhn, Sereina M. Graber, Thomas A. Neff, Martin Schläpfer

**Affiliations:** 1 Institute of Anesthesiology, University Hospital Zurich, Raemistrasse 100, Zurich, 8091, Switzerland; 2 Institute of Physiology, Zurich Center for Integrative Human Physiology, University of Zurich, Winterthurerstrasse 190, Zurich 8057, Switzerland; 3 Department of Anesthesiology, University of Illinois College of Medicine at Chicago, 1740 West Taylor Street, Suite 3200 West, Chicago, IL, 60612, United States of America; 4 Antropological Institute and Museum, University of Zurich, Winterthurerstrasse 190, Zurich, 8057, Switzerland; 5 Department of Anesthesia & Intensive Care Medicine, Cantonal Hospital of Muensterlingen, Campus 1, Muensterlingen, 8596, Switzerland; University of Louisville, UNITED STATES

## Abstract

**Introduction:**

In a recent randomized controlled trial our group has demonstrated in 102 patients that late post-conditioning with sevoflurane performed in the intensive care unit after surgery involving extracorporeal circulation reduced damage to cardiomyocytes exposed to ischemia reperfusion injury. On the first post-operative day the sevoflurane patients presented with lower troponin T values when compared with those undergoing propofol sedation. In order to assess possible clinical relevant long-term implications in patients enrolled in this study, we performed the current retrospective analysis focusing on cardiac and non-cardiac events during the first 6 months after surgery.

**Methods:**

All patients who had successfully completed the late post-conditioning trial were included into this follow-up. Our primary and secondary endpoints were the proportion of patients experiencing cardiac and non-cardiac events, respectively. Additionally, we were interested in assessing therapeutic interventions such as initiation or change of drug therapy, interventional treatment or surgery.

**Results:**

Of 102 patients analyzed in the primary study 94 could be included in this follow-up. In the sevoflurane group (with 41 patients) 16 (39%) experienced one or several cardiac events within 6 months after cardiac surgery, in the propofol group (with 53 patients) 19 (36%, p=0.75). Four patients (9%) with sevoflurane vs. 7 (13%) with propofol sedation had non-cardiac events (p=0.61). While a similar percentage of patients suffered from cardiac and/or non-cardiac events, only 12 patients in the sevoflurane group compared to 20 propofol patients needed a therapeutic intervention (OR: 0.24, 95% CI: 0.04-1.43, p=0.12). A similar result was found for hospital admissions: 2 patients in the sevoflurane group had to be re-admitted to the hospital compared to 8 in the propofol group (OR 0.23, 95% CI: 0.04-1.29, p=0.10).

**Conclusions:**

Sevoflurane does not seem to provide protection with regard to the occurrence of cardiac and non-cardiac events in the 6-month period following cardiac surgery with the use of extracorporeal circulation. However, there was a clear trend towards fewer interventions (less need for treatment, fewer hospital admissions) associated with sevoflurane post-conditioning in patients experiencing any event. Such results might encourage launching large multicenter post-conditioning trials with clinical outcome defined as primary endpoint.

## Introduction

Strategies to minimize ischemia—reperfusion injury have been a topic of discussion over decades [1]. Conditioning of the ischemic organ can occur at different time points in relation to the ischemic insult: before the onset of ischemia (pre-conditioning) [2, 3], after the onset of ischemia (post-conditioning) [4] or throughout the ischemic event (per-conditioning) [5, 6]. The nature of the intervention can be mechanical (several brief periods of ischemia followed by reperfusion and re-oxygenation), or pharmacological. Both mechanisms seem to share common pathways and different mediators play a role at cellular and subcellular level, primarily providing mitochondrial protection from the ischemic insult [7, 8].

Modern volatile anesthetics such as sevoflurane or desflurane have proven efficient in reducing ischemia reperfusion injury in various settings (pharmacological conditioning). In liver surgery both pre- and post-conditioning strategies lead to a significant reduction of hepatocellular injury as well as perioperative complications [9, 10]. In heart surgery procedures involving extracorporeal circulation (ECC), exposing myocardial tissue to ischemia-reperfusion induces cardiomyocyte damage, possibly leading to perioperative infarction, increasing morbidity and mortality for up to three years [11, 12]. In this setting the use of volatile anesthetics leads to reduced myocardial tissue damage, a reduction of myocardial infarction and overall mortality [8, 13–15].

In a recent randomized controlled trial (RCT) our group has demonstrated that patients could benefit from exposure to volatile anesthetics after heart surgery as late as upon arrival in the intensive care unit (ICU, late post-conditioning) [16]. In that study 117 patients scheduled for elective cardiac surgery requiring the use of ECC at the University Hospital Zurich, Switzerland, were sedated with target-controlled propofol infusion during the surgical procedure. After arrival at the ICU, sedation was continued for at least 4 hours either with propofol or, defined as the alternative intervention, with sevoflurane. Late post-conditioning with sevoflurane proved to be superior to propofol, reducing myocardial injury measured by the specific surrogate biochemical marker troponin T at post-operative day 1.

For sevoflurane pre-conditioning, an early (first 23h after pre-conditioning) and a late (12-96h after pre-conditioning) window have been described before, with the latter protecting patients undergoing coronary artery bypass grafting from late cardiac events [11]. In analogy to this study of Garcia et al. [11] and in order to assess possible clinically relevant long-term implications of late sevoflurane post-conditioning in patients enrolled in our primary study, we decided to perform a retrospective analysis, in which we focused on cardiac as well as on non-cardiac events and their diagnostic and/or therapeutic consequences over a period of 6 months following the surgical procedure. We hypothesized that sevoflurane patients might have a better outcome.

## Materials and Methods

The RCT “Late pharmacologic conditioning with volatile anesthetics after cardiac surgery” was published in Critical Care in October 2012 [16]. This study was approved by the local ethics committee (Kantonale Ethikkommission, Zurich, Switzerland, trial number StV 5–2007) and was registered in ClinicalTrials.gov (NCT00924222).

In the RCT, conducted between October 2007 and September 2009 at the University Hospital Zurich, Switzerland, 102 patients undergoing cardiopulmonary bypass for aortic or mitral valve surgery with or without coronary bypass grafting were included. Potential short-term benefits of late post-conditioning for ≥4h upon arrival at the ICU comparing propofol and sevoflurane sedation were evaluated. Patients had to be between 18 and 90 years old. Exclusion criteria were poor cardiac baseline function, significant coronary impairment, emergency procedures, previous cardiac surgery, chronic pulmonary disease, renal dysfunction, insulin-dependent diabetes mellitus, pregnancy, and current steroid treatment. Randomization took place by a computer. Both, the surgeon and the anesthesiologist in the operating theatre were blinded to the intervention group. The primary endpoint of this study was cardiac injury on postoperative day 1 (POD1), measured by cardiac injury markers. Secondary endpoints included oxygenation index after 4 hours of sedation, incidence of postoperative pulmonary complications, and the need for antiemetic drugs.

The main finding was a decreased troponin T level on POD1 in patients sedated with sevoflurane in comparison to patients sedated with propofol.

### Evaluation of the 6-month follow-up

This retrospective study was approved by the ethical committee of the canton of Zurich, Switzerland (KEK-ZH No. 2014–0040, April 22^nd^, 2014) and was registered at clinicaltrials.gov (NCT02378168).

All patients who had successfully completed the late post-conditioning trial [16] were included into this retrospective follow-up analysis. We obtained written and informed consent of all patients for the first study. No consent had been withdrawn either before or after publication of the first trial. According to the ethical committee, no new consent had to be obtained. Patient data were anonymized prior to further analysis.

The time frame of our retrospective analysis was limited to 6 months after surgery. Data were retrieved primarily from the internal hospital database. In addition, secondary care units and family doctors were contacted by sending them a concise questionnaire. To assure data completeness these institutions were, when necessary, contacted by phone by one of our consultant anesthesiologist between May 2014 and August 2014. Researchers gathering data were blinded for the study group of the patients.

The primary endpoint was the proportion of patients with cardiac events (dysrhythmias, congestive heart failure, and cardiac ischemic event). We also assessed re-admission to the hospital and therapeutic interventions such as introduction or modification of specific drug regimen, interventional treatments such as cardioversion or catheter based treatments (e.g. thermal ablation of atrial flutter, percutaneous coronary intervention), as well as conventional surgery (e.g. coronary artery bypass grafting, Maze procedure, Batista procedure).

The proportion of patients experiencing non-cardiac events was defined as the secondary endpoint. We focused on acute or chronic pulmonary embolism, bleeding events, infections, cerebral events, and acute as well as chronic kidney failure, defined as glomerular filtration rate below 60 ml/min. In line with the primary endpoints we assessed the necessity for re-admission to the hospital, for drug or interventional treatment (e.g. cerebral angiography with thrombectomy or intra-arterial lysis, dialysis), and the need for surgery (e.g. surgical hemostasis, surgical abscess evacuation, and kidney transplantation).

If any question (primary or secondary endpoints) was answered affirmatively, family doctors were asked to provide detailed information about the event, indicating how exactly patients had to be treated for the event and whether they had to be re-admitted to the hospital. Details about the patient included into this study are found in S1 Dataset.

Statistical analysis of the anonymized data was performed using Programm R (R Development Core Team, R Foundation for Statistical Computing, Vienna, Austria) and GraphPad Prism 6 (GraphPad Software Inc, La Jolla, CA). Logistic regression models with propofol as reference group were chosen. Odds ratio (OR), 95% confidence interval (95% CI), and p-values were computed. Patient characteristics were evaluated by Mann-Whitney and Fisher’s exact tests. For bootstrapping we used an ordinary nonparametric approach based on 10,000 replications. A p-value of <0.05 was considered significant.

### Power estimation

The maximal number of patients available for this study was given by the primary randomized controlled trial, in which 102 patients were included. A power calculation based on a Chi-Square test with a power of 90%, an estimated effect size of 10%, and an alpha-error of 0.05 would result in a total sample size of 964 individuals. The power issue is discussed in detail in the discussion section.

## Results

### Patient enrollment

**Fig 1:**
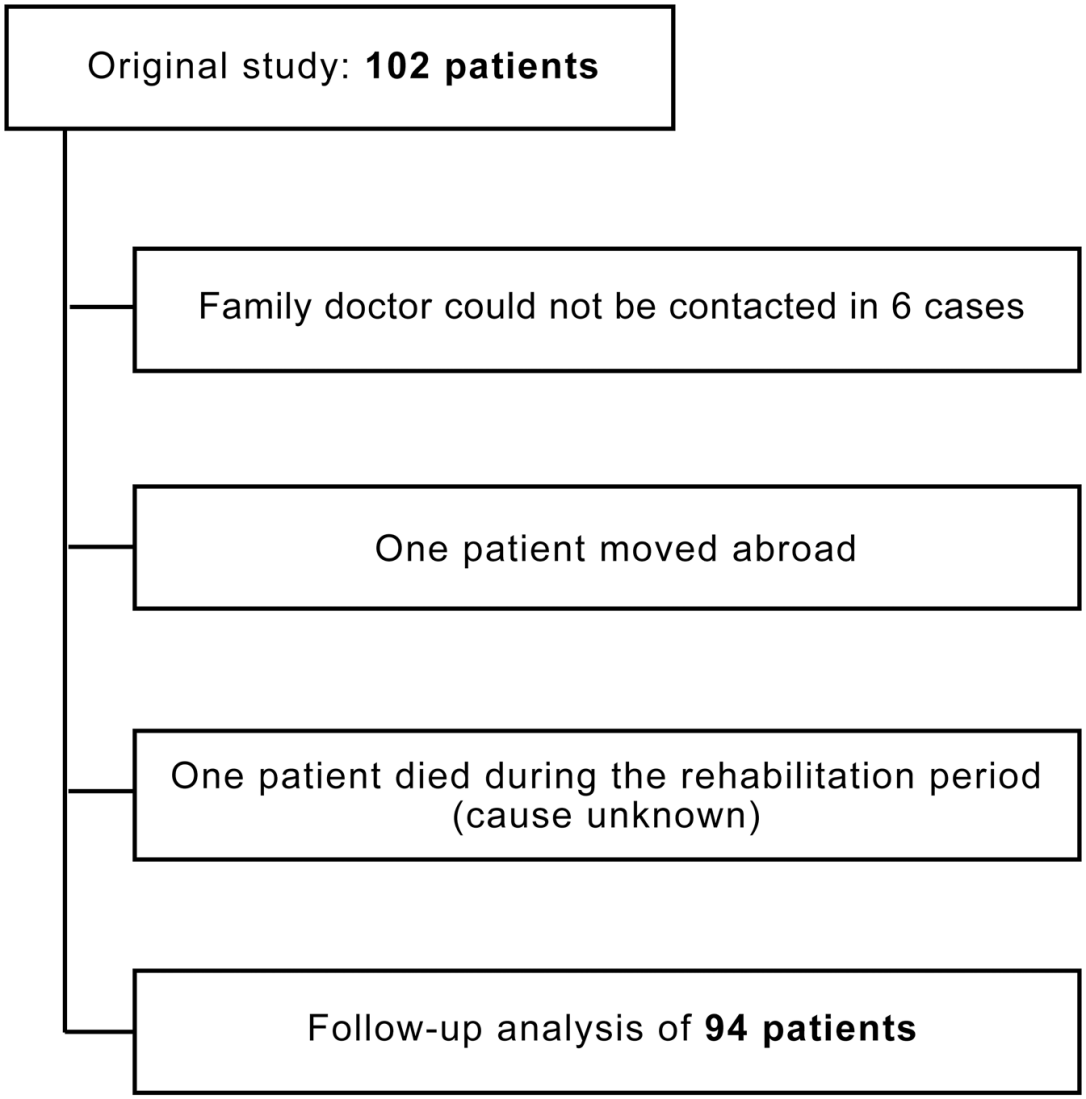
Included patients. Flow chart of the patients included into the late post-conditioning study and patients that could be enrolled to the six-month follow-up study after late cardiac surgery.

Of the 102 patients analyzed in the trial of Steurer et al. [16], 94 could be included in this follow-up study. For 6 patients there were no data in the hospital database available, nor could the family doctor be contacted. One patient had left the country after hospitalization, while another had died during his rehabilitation period. Unfortunately, no information was available about the circumstances of this death. Therefore, this patient was listed under ‘characteristics’ (n = 95, Table 1), but due to missing information not taken into account for the detailed analysis of adverse events during the follow-up period (n = 94) (Fig 1).

**Table 1:** Demographic and surgical characteristics.

	sevoflurane	propofol	p-value
	n = 42	n = 53	
Age (years), median (25%-75% percentile)	66 (54–73)	67 (54–75)	0.65
Male subjects, n (%)	27 (64)	39 (74)	0.37
Aortic valve surgery, n (%)	35 (83)	42 (79)	0.79
Isolated mitral valve surgery, n (%)	6 (14)	11 (21)	0.59
Concurrent aortocoronary bypass surgery, n (%)	13 (31)	17 (32)	1.00

Data from 60% (56 out of 94) of the patients could be gathered from our hospital information system and were compared to the data provided from family doctors. While 89% (41/46) of the data were complete in the sevoflurane group, the according number was 95% (53/56) in the propofol group.

### Demographic and surgical characteristics

Patient characteristics were similar in the sevoflurane and the propofol group as expected in an RCT. Median age after 6-month follow-up was 66 (25%-75% percentile: 54–73) years

in the sevoflurane and 67 (25%-75% percentile: 54–75) years in the propofol group. The sevoflurane group consisted of 27 (64%) male subjects, the propofol group of 39 (74%) (Table 1).

Of the 94 patients analyzed, 77 patients (82%) underwent aortic valve surgery, 35 (45%) in the sevoflurane group and 42 (55%) in the propofol group. Concurrent aortocoronary bypass was similar in both groups: sevoflurane 13 (31%) and propofol 17 (32%). Isolated mitral valve surgery was performed in 17 patients (18%), 6 (14%) in the sevoflurane group and 11 (21%) in the propofol group.

### Patients with cardiac events

In the sevoflurane group (n = 41) 16 patients (39%), in the propofol group (n = 53) 19 (36%) suffered from one or several cardiac events during the first 6 months after cardiac surgery. Table 2 gives an overview of patients with reported events and the corresponding therapies initiated. Of the 16 sevoflurane patients 4 patients experienced two events, of the 19 propofol patients 5 patients had two and one patient three cardiac events.

**Table 2:** Patients with cardiac events: type of event, therapies initiated and need for hospitalization.

	sevoflurane	propofol	OR	95% CI		p-value
total number of patients (% of all patients)	16 (39)	19 (36)	1.15	0.49	2.66	0.75
patients with dysrhythmias (%)	13 (32)	12 (23)	1.59	0.63	3.98	0.33
drug or interventional treatment, n	11	13				
surgery, n	1	1				
admission to hospital, n	2	3				
patients with congestive heart failure (%)	7 (17)	13 (25)	0.63	0.23	1.77	0.38
drug or interventional treatment, n	7	13				
surgery, n	0	1				
admission to hospital, n	0	1				
patients with cardiac ischemia (%)	0 (0)	1 (2)	0.00	0.00	inf.	1.00
drug or interventional treatment, n	0	1				
surgery, n	0	0				
admission to hospital, n	0	0				

OR: odds ratio

CI: confidence interval

inf.: infinite.

The chance for a patient to have a cardiac event was similar in both groups (overall OR: 1.15; 95% CI: 0.49–2.66, p = 0.75, Table 2). The most common cardiac event was dysrhythmia, with an OR of 1.59 to occur in the sevoflurane group (95% CI: 0.63–3.98, p = 0.33, Table 2), followed by congestive heart failure. Also here, no statistical significance could be reached comparing the two patient groups. Of note is that chances were smaller to experience congestive heart failure in the sevoflurane compared to the propofol group (OR: 0.63, 95% CI: 0.23–1.77, p = 0.38, Table 2).

For dysrhythmias, being the most common cardiac complication, the rate for intervention and surgery was similar in both groups: Nine of 13 patients in the sevoflurane group vs. 11 of 12 propofol patients had to undergo drug treatment for dysrhythmias (OR: 0.21, 95% CI: 0.02–2.17, p = 0.19, Table 2).

Congestive heart failure was diagnosed in 7 patients in the sevoflurane group and 13 in the propofol group (OR: 0.63, 95% CI: 0.23–1.77, p = 0.38, Table 2). All these patients underwent drug and/or interventional treatment.

One patient in the propofol group suffered from cardiac ischemia and had to undergo interventional treatment (Table 2).

### Patients with non-cardiac events

**Fig 2:**
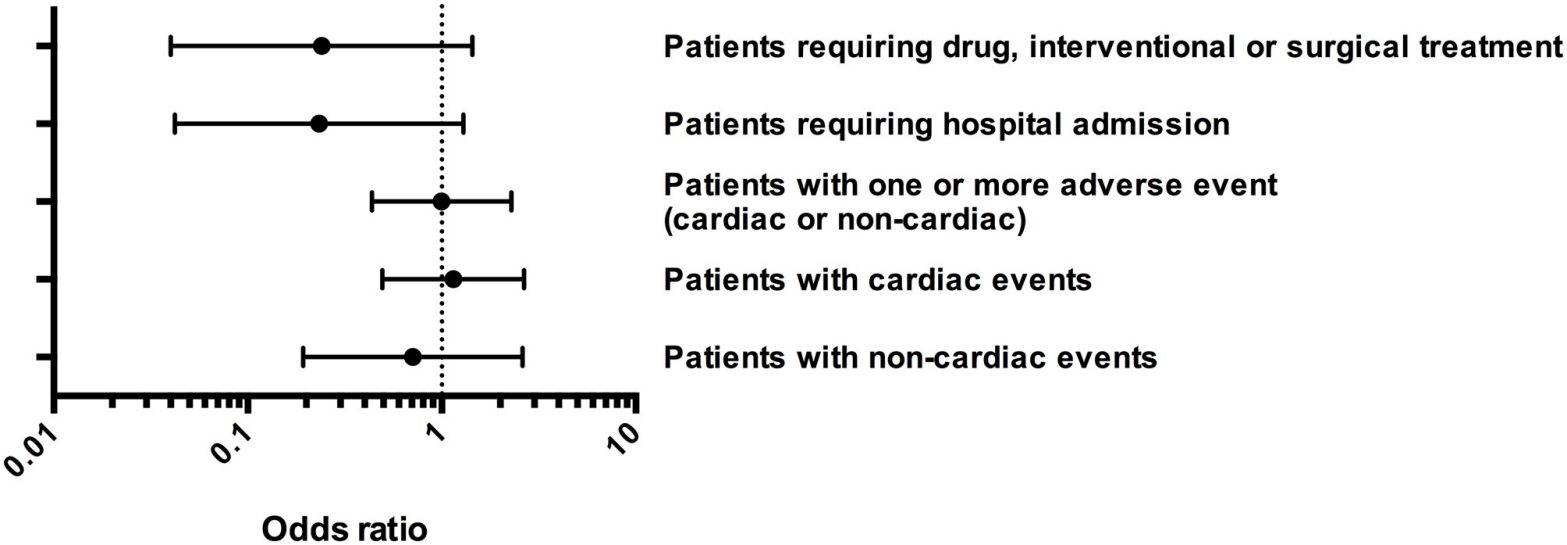
Type of sedation and odds ratio for adverse events. Odds ratios and 95% confidence intervals of patients with overall adverse events, with cardiac and non-cardiac events, with need for drug, interventional or surgical treatment and need for admission to the hospital. Patients with propofol sedation in the ICU were taken as the reference group.

Non-cardiac events (pulmonary embolism, bleeding, infections, cerebral events, acute and chronic kidney failure) were reported in 4 (9%) patients with sevoflurane vs. 7 (13%) with propofol sedation. The OR for a patient in the sevoflurane group suffering from a non-cardiac complication was 0.71 without reaching statistical significance (95% CI: 0.19–2.61, p = 0.61, Table 3, Fig 2). All 4 sevoflurane patients had only one event, whereas in the propofol group 2 patients had 2 events.

**Table 3:** Patients with non-cardiac events: type of event, therapies initiated and need for hospitalization.

	sevoflurane	propofol	OR	95% CI		p-value
total number of patients (% of all patients)	4 (9)	7 (13)	0.71	0.19	2.61	0.61
pulmonary embolism, n (%)	0 (0)	1 (2)	0.00	0.00	inf.	1.00
drug or interventional treatment, n	0	1				
surgery, n	0	0				
admission to hospital, n	0	1				
bleeding, n (%)	0 (0)	2 (4)	0.00	0.00	inf.	1.00
drug or interventional treatment, n	0	1				
surgery, n	0	1				
admission to hospital, n	0	2				
infection, n (%)	3 (8)	2 (4)	2.01	0.32	12.65	0.46
drug or interventional treatment, n	1	0				
surgery, n	0	1				
admission to hospital, n	0	2				
cerebral events, n (%)	1 (3)	0 (0)	>100	0.00	inf.	1.00
drug or interventional treatment, n	1	0				
surgery, n	0	0				
admission to hospital, n	0	0				
chronic kidney failure, n (%)	0 (0)	4 (8)	0.00	0.00	inf.	1.00
drug or interventional treatment, n	0	1				
surgery, n	0	0				
admission to hospital, n	0	2				

OR: odds ratio

CI: confidence interval

inf.: infinite.

Three patients in the sevoflurane group experienced an infection, one of them needing an intervention. In addition, a cerebral event was reported in one patient. In the propofol group 2 patients were diagnosed with pulmonary embolism, 2 had bleeding events, 2 were identified with infections and 4 with chronic kidney failure (Table 3). None of these differences were significant.

### Combination of patients experiencing cardiac and non-cardiac events

Of the 94 patients analyzed, a similar number of patients in the sevoflurane (17 patients, 41%) and in the propofol group (22 patients, 42%) suffered from one or several adverse events (cardiac and/or non-cardiac) with an OR of 1.00 (95% CI: 0.44–2.28, p = 1.00, Fig 2, Table 4). As some patients have experienced a cardiac as well as a non-cardiac event the total number of patients in the according group (sevoflurane n = 17, propofol n = 22) is smaller than the sum of the patients with cardiac (sevoflurane n = 16, propofol n = 19) and non-cardiac events (sevoflurane n = 4, propofol n = 7). Six patients in the sevoflurane group and 7 patients in the propofol group were reported with two or more events.

**Table 4:** Patients with cardiac and/ or non-cardiac events, need for treatment and need for hospitalization.

	sevoflurane	propofol	OR	95% CI		p-value
total number of patients (% of patients with events)	17 (44)	22 (56)	1.00	0.44	2.28	1.00
drug, interventional treatment or surgery, n (%)	12 (31)	20 (51)	0.24	0.04	1.43	0.12
admission to hospital, n (%)	2 (5)	8 (21)	0.23	0.04	1.29	0.10

OR: odds ratio

CI: confidence interval.

Interestingly, despite a similar number of patients with adverse events, only 12 patients (31% of the patients with an adverse event) in the sevoflurane group compared to 20 (51%) in the propofol group needed a drug, an interventional treatment, and/or surgery (OR: 0.24, 95% CI: 0.04–1.43, p = 0.12, Fig 2, Table 4). Bootstrapping based on 10,000 replications did lead to similar results (median OR: 0.22, 95% CI: 0.00–4.00). A similar picture was observed for hospital admissions: 2 sevoflurane patients (5% of the patients with ≥1 event) as compared to 8 propofol patients (21% of the patients with ≥1 event) had to be re-admitted to the hospital (OR 0.23, 95% CI: 0.04–1.29, p = 0.10, Fig 2, Table 4). Also for this data the odds ratio could be confirmed by bootstrapping (median OR: 0.21, 95% CI: 0.00–2.21).

## Discussion

In a recent randomized controlled trial from our group [16], immediate cardioprotective effects of late anesthetic post-conditioning with sevoflurane after aortic and mitral valve replacement in combination with CABG or ascending aorta surgery have been reported. The current follow-up study has investigated the potential impact of sevoflurane exposure on a 6-month long-term outcome with regard to cardiac and non-cardiac complications. While no statistical significance was reached, the results clearly point towards a possible beneficial effect of sevoflurane post-conditioning. Data from this follow-up study suggest a 4 times lower risk for the *need for medical treatment* (12 vs. 20 patients; OR 0.240; 95% CI 0.040–1.43; p = 0.118) and *need for hospital admission* (2 vs. 8 patients; OR 0.233, 95% CI: 0.042–1.293; p = 0.095, Table 4) in the sevoflurane as compared to the propofol group when experiencing a cardiac and/or non-cardiac event. The observed differences in the *need for medical treatment* could be regarded as composite endpoint for future studies. In order to address the problem of a potential bias and model assumption violations bootstrap analyses were performed, which could further underline a certain robustness of the data. Even bootstrap results support the 6-months follow-up data, this procedure of course does not overcome the problem of a potential lack of power.

An estimated 4% of the worldwide population undergoes major surgery requiring regional or general anesthesia each year [17]. Overall, the incidence of postoperative complications and death is low. However, sub-groups of high-risk patients (e.g. advanced age, co-morbid disease, major, and urgent surgery) are associated with increased risk for complications. Given the substantial costs associated with major postoperative complications, reducing morbidity by innovative therapies may be essential to economize health care resources, to reduce overall cost of care, and to improve quality of care.

It is well known from extensive clinical [11, 14, 15, 18–23] and experimental work [8, 24–29] that halogenated anesthetics may exert many non-anesthetic properties such as inducing an endogenous adaptive response of cells against ischemia-reperfusion injury [30, 31]. Protection is thereby mediated by adenosine receptor stimulation with subsequent activation of protein kinase C, increased production of nitric oxide, and free oxygen [32].

In non heart beating surgery, myocardial ischemia and reperfusion is an inherent and inevitable part of the surgical procedure itself. Strategies capable of limiting the ischemic trauma of cardiomyocytes and thus improving cardiac recovery are therefore of pivotal interest. Conservation of postoperative cardiac function was first described in 1999 in a small clinical study that used a pre-conditioning protocol with isoflurane before cross-clamping of the aorta [18]. A meta-analysis from Landoni and co-workers in 2007 suggested that sevoflurane and desflurane are equally effective in reducing the incidence of myocardial infarction and postoperative mortality after cardiac surgery [13].

Beyond well-described immediate protective effects of volatile anesthetics on specific and organ dependent biomarkers in various clinical and experimental settings, it is of major interest whether these results could be translated from the level of surrogate markers to clinical outcome after cardiac surgery. Some studies have already shown certain benefits of volatile anesthetics on clinical parameters such as duration of mechanical ventilation, length of ICU as well as hospital stay [15, 23, 33, 34]. Both, reduction of ventilator time as well as length of stay are considered relevant in terms of patient recovery and cost. A recent randomized controlled trial from our group [10] clearly demonstrated that pharmacological post-conditioning with sevoflurane in patients undergoing liver resection not only significantly reduced peak levels of aspartate transaminase (AST), a valuable biomarker for liver injury, it also had a significant impact on the occurrence of postoperative complications, determined by a the well-established Clavien-Dindo scoring system [35].

Immediate cardioprotective effects of late anesthetic post-conditioning with sevoflurane after aortic and mitral valve replacement in combination with coronary artery bypass surgery (CABG) or ascending aorta surgery revealed a benefit for the sevoflurane group, reflected in a lower cardiac damage, measured by the surrogate marker troponin T, on POD 1 [16] whereas this follow-up study has investigated the potential impact of sevoflurane exposure on a 6-month long-term outcome with regard to clinical cardiac and non-cardiac complications. In the primary trial baseline characteristics of the two groups were similar [16] as expected with an RCT design minimizing therefore the possibility of unbalanced group bias on the troponin T differences.

The relevance of troponin T on long-term outcome after cardiac surgery is not quite clear. Brown and colleagues could not associate troponin T levels after on- and off-pump coronary artery bypass with a prognostic value [36]. However recent studies and meta-analyses did associate high troponin T levels with poor long-term outcome after vascular surgery [37], after percutaneous coronary interventions [38], and after cardiac transplantation [39].

The lack of significance in this study may be explained by the fact that the initial sample size calculation was performed for the primary study and was targeting the acute cardiac injury markers troponin, creatine kinase and myoglobin. In contrast to the primary study, the endpoints of the actual follow-up study were designed to detect differences in pure clinical endpoints: the appearance of cardiac and non-cardiac events with diagnostic and/or therapeutic consequences including hospital re-admission within a 6-month follow-up period after surgery. Hence, the follow-up study is likely to be underpowered for these respective endpoints. If we would calculate the sample size of the groups based on the endpoints we focused on in this follow-up study we would have needed 7302 patients in our analysis to detect a difference in postoperative cardiac events. To detect differences in the *need for medical treatment* (drug therapy, interventional treatment or surgery) already 1036 patients would have been sufficient, and for detecting a difference in *hospital admission rates* significance would have been reached with a total of 160 patients. The above mentioned power calculations are based on a Chi-square test with a power of 80%, an alpha-error of 0.05 and using the results of our follow-up study for the proportions.

Several limitations of the study have to be acknowledged. First, the post-conditioning phase was relatively short with 4 hours only. Second, the applied dose of sevoflurane reflecting an age-adjusted 0.5 minimum alveolar concentration (MAC) was rather low when compared to anesthetic concentrations during surgery. Higher concentrations of volatile anesthetics and prolonged exposure might have exerted more pronounced organ-protection and turned the results into significance.

In addition, propofol may also be able to offer protective effects, especially via ATP sensitive potassium channels [40]. It appears to act synergistic with volatile anesthetic conditioning [41]. When addressing potential effects of propofol a word of caution should be warranted as it is clinically available as emulsified solution of drug and additives. The presence of ethylenediaminetetraacetic acid (EDTA) is particularly relevant since EDTA itself offers anti-inflammatory properties by destabilizing bacterial walls [42]. Therefore it might be difficult to separate effects of the drug from those of the additives.

The major strength of this study is its data quality. Even though data collection was done in a retrospective manner, data robustness is very high since the follow-up was based on a randomized controlled trial. In addition, a carefully developed and standardized questionnaire was used for data retrieval. The return rate of 93% of questionnaires was exceptionally high. Another remarkable aspect of our study is the exclusive use of clinical follow-up parameters, which might be more relevant than studying biochemical surrogate markers of uncertain clinical significance.

Despite the lacking significance of our data, promising trends are reported, which might translate in reduced postoperative adverse events and reduced treatment cost. This emphasizes the importance of collecting clinical outcome data on this important topic in the future. Larger randomized trials will add more value to the current body of evidence and are highly likely to reveal an improved clinical outcome by exposure to volatile anesthetics.

## Conclusions

We document a similar percentage of cardiac as well as of non-cardiac events in patients exposed to sevoflurane as compared to propofol in a post-conditioning manner. Despite not reaching statistical significance, we observed less severe complications in the sevoflurane group (less need for treatment, fewer hospital readmissions). Statistical significance might not have been reached because the original study was powered for biochemical surrogate markers and not for clinical outcomes.

## Supporting Information

S1 Dataset Dataset of the patients included into the study. (XLSX)
